# CAF secreted miR-522 suppresses ferroptosis and promotes acquired chemo-resistance in gastric cancer

**DOI:** 10.1186/s12943-020-01168-8

**Published:** 2020-02-27

**Authors:** Haiyang Zhang, Ting Deng, Rui Liu, Tao Ning, Haiou Yang, Dongying Liu, Qiumo Zhang, Dan Lin, Shaohua Ge, Ming Bai, Xinyi Wang, Le Zhang, Hongli Li, Yuchong Yang, Zhi Ji, Hailong Wang, Guoguang Ying, Yi Ba

**Affiliations:** Tianjin Medical University Cancer Institute and Hospital, National Clinical Research Center for Cancer, Tianjin’s Clinical Research Center for Cancer, Key Laboratory of Cancer Prevention and Therapy, Tianjin, 300060 China

**Keywords:** Ferroptosis, Cancer-associated fibroblasts, Exosomes, miR-522, GC

## Abstract

**Background:**

Ferroptosis is a novel mode of non-apoptotic cell death induced by build-up of toxic lipid peroxides (lipid-ROS) in an iron dependent manner. Cancer-associated fibroblasts (CAFs) support tumor progression and drug resistance by secreting various bioactive substances, including exosomes. Yet, the role of CAFs in regulating lipid metabolism as well as ferroptosis of cancer cells is still unexplored and remains enigmatic.

**Methods:**

Ferroptosis-related genes in gastric cancer (GC) were screened by using mass spectrum; exosomes were isolated by ultra-centrifugation and CAF secreted miRNAs were determined by RT-qPCR. Erastin was used to induce ferroptosis, and ferroptosis levels were evaluated by measuring lipid-ROS, cell viability and mitochondrial membrane potential.

**Results:**

Here, we provide clinical evidence to show that arachidonate lipoxygenase 15 (ALOX15) is closely related with lipid-ROS production in gastric cancer, and that exosome-miR-522 serves as a potential inhibitor of ALOX15. By using primary stromal cells and cancer cells, we prove that exosome-miR-522 is mainly derived from CAFs in tumor microenvironment. Moreover, heterogeneous nuclear ribonucleoprotein A1 (hnRNPA1) was found to mediate miR-522 packing into exosomes, and ubiquitin-specific protease 7 (USP7) stabilizes hnRNPA1 through de-ubiquitination. Importantly, cisplatin and paclitaxel promote miR-522 secretion from CAFs by activating USP7/hnRNPA1 axis, leading to ALOX15 suppression and decreased lipid-ROS accumulation in cancer cells, and ultimately result in decreased chemo-sensitivity.

**Conclusions:**

The present study demonstrates that CAFs secrete exosomal miR-522 to inhibit ferroptosis in cancer cells by targeting ALOX15 and blocking lipid-ROS accumulation. The intercellular pathway, comprising USP7, hnRNPA1, exo-miR-522 and ALOX15, reveals new mechanism of acquired chemo-resistance in GC.

**Graphical abstract:**

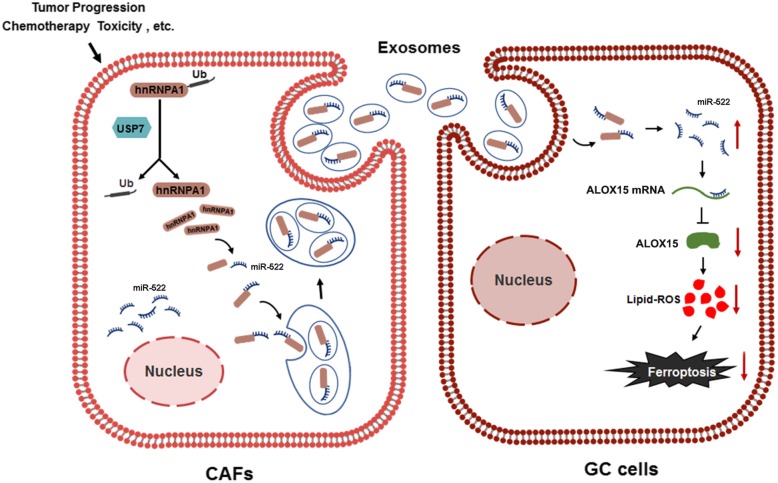

## Introduction

Cell death is strictly regulated by complex intracellular and extracellular signals, and is essential for various biological processes, including homeostasis, development and disease. The imbalance between proliferation and death of cancer cells is the important basis that leads to malignant biological characteristics. Cancer cells have shown complicated strategies of metabolic adaptation to survive under metabolic stress conditions and to allow tumor progression, including blocking of apoptosis, as well as non-apoptotic cell death pathways [1, 2]. Ferroptosis, a novel form of regulated cell death, involves iron-dependent lipid peroxides (lipid-ROS) accumulation and leads to lethal damage of cells [3]. Recent studies have identified the essential role of ferroptosis in mediating tumor development and drug-resistance in some types of cancer, but the detailed molecular mechanism remains poorly understood [4–7].

Tumor microenvironment (TME) consists of extracellular matrix and mesenchymal cell types, including fibroblasts, inflammatory cells, pericytes and endothelial cells [8]. Cancer-associated fibroblasts (CAFs) are the main type of stromal cells in tumor microenvironment and exhibit distinct tumorigenic properties [9–11]. CAFs, as well as the other tumor stromal cells, surrounding the primary foci generate signals or factors to regulate tumor phenotypes, displaying the capacity to influence each stage of tumor development [12, 13].

Exosomes belong to the family of extracellular vesicles (EVs) with a typical diameter of 30–100 nm, and are secreted by most cell types [14]. In the past decade, exosomes have been treated as the novel message transmitters in intercellular communication by delivering proteins, lipids, lncRNAs, circRNAs and microRNAs [15, 16]. Recent studies indicated that exosomes derived from CAFs promote tumor metastasis and increase chemo-resistance of cancer cells [17, 18]. Exosome-miR-21 derived from CAFs was found to be involved in oxaliplatin resistance in colorectal cancer [19]; and CAF-secreted exo-miR-146a are proved to regulate survival and proliferation of pancreatic cancer cells [20]. However, the potential roles of exosomes derived from CAFs in regulating lipid metabolism and ferroptosis in cancer cells have not been defined yet.

In this study, ferroptosis is found to be significantly inhibited in gastric cancer (GC), contributing to tumor growth and decreased sensitivity to cisplatin and paclitaxel. And arachidonate lipoxygenase 15 (ALOX15), the main mediator of lipid-ROS production in GC cells, is observably down-regulated and is found to be closely linked with the suppression of ferroptosis. Moreover, exosomal miR-522 secreted from CAFs plays a dominant role in regulating ALOX15 expression in GC cells. Additionally, we demonstrated that ubiquitin-specific protease 7 (USP7) and recombinant human heterogeneous nuclear ribonucleoprotein A1 (hnRNPA1) are involved in the process of miR-522 sorting into exosomes. Therefore, this study identifies a novel network mediated by exosomes that regulates ferroptosis of cancer cells, and provides novel methods to enhance chemo-sensitivity of gastric cancer.

## Methods

### Human tissues

The tumor tissue samples and plasma samples of GC patients were obtained from Tianjin Medical University Cancer Institute and Hospital.

### Animals

Male nude mice (BALB/c-nu, 6B8 weeks) were housed in a pathogen free animal facility with access to water and food, and allowed to eat and drink adlibitum.

### Cell culture

Human gastric cancer cell lines, SGC7901 (human gastric adenocarcinoma cell), MGC803 cells and MKN45 cells were bought from cell bank of Chinese Academy of Sciences (Shanghai, China), and were cultured in DMEM medium (Gibco, USA) supplemented with 10% fetal bovine serum. Each cell line was tested for mycoplasma contamination before use.

### Isolation of exosomes from medium and plasma

Exosomes in medium and plasma were isolated from cell by differential centrifugation, according to previous publications [21]. After removing cells and other debris by centrifugation at 300 g and 3000 g respectively, the supernatant was centrifuged at 10,000 g for 30 min to remove shedding vesicles and the other vesicles with larger sizes. Finally, the supernatant was centrifuged at 110,000 g for 70 min, and exosomes were collected from the pellet and re-suspended in PBS (all steps were performed at 4 °C).

### Transmission electron microscopy assay (TEM)

For conventional TEM, the exosome pellet was placed in a droplet of 2.5% glutaraldehyde in PBS buffer at pH 7.2 and fixed overnight at 4 °C. Samples were rinsed in PBS buffer (3 times, 10 min each) and post-fixed in 1% osmium tetroxide for 60 min at room temperature. The samples were then embedded in 10% gelatin and fixed in glutaraldehyde at 4 °C and cut into several blocks (less than 1 mm3). The samples were dehydrated for 10 min each step in increasing concentrations of alcohol (30, 50, 70, 90, 95, and 100% × 3). Pure alcohol was then exchanged by propylene oxide, and specimens were infiltrated with increasing concentrations (25, 50, 75, and 100%) of Quetol-812 epoxy resin mixed with propylene oxide for a minimum of 3 h per step. Samples were embedded in pure, fresh Quetol-812 epoxy resin and polymerized at 35 °C for 12 h, 45 °C for 12 h, and 60 °C for 24 h. Ultrathin sections (100 nm) were cut using a Leica UC6 ultra-microtome and post-stained with uranyl acetate for 10 min and with lead citrate for 5 min at room temperature before observation in a FEI Tecnai T20 transmission electron microscope, operated at 120 kV.

### Nanoparticle tracking analysis (NTA)

The size and density of exosomes were directly tracked using the Nanosight NS 300 system (NanoSight technology, Malvern, UK) [22, 23]. Exosomes were re-suspended in PBS at a concentration of 5 μg/mL were further diluted 100- to 500-fold to achieve between 20 and 100 objects per frame. Samples were manually injected into the sample chamber at ambient temperature. Each sample was configured with a 488 nm laser and a high-sensitivity sCMOS camera, and was measured in triplicate at camera setting 13 with an acquisition time of 30s and a detection threshold setting of 7. At least 200 completed tracks were analyzed per video. Finally, data was analyzed using the NTA analytical software (version 2.3).

### Mass Spectrum analysis

LC-MS/MS analysis was performed on a Q Exactive mass spectrometer (Thermo Scientific) that was coupled to Easy nLC (Proxeon Biosystems, now Thermo Fisher Scientific) for 60/120/240 min (determined by project proposal). The mass spectrometer was operated in positive ion mode. MS data was acquired using a data-dependent top10 method dynamically choosing the most abundant precursor ions from the survey scan (300–1800 m/z) for HCD fragmentation. Automatic gain control (AGC) target was set to 3e6, and maximum inject time to 10 ms. Dynamic exclusion duration was 40.0 s. Survey scans were acquired at a resolution of 70,000 at m/z 200 and resolution for HCD spectra was set to 17,500 at m/z 200, and isolation width was 2 m/z.. Normalized collision energy was 30 eV and the underfill ratio, which specifies the minimum percentage of the target value likely to be reached at maximum fill time, was defined as 0.1%. The instrument was run with peptide recognition mode enabled. The MS data were analyzed using MaxQuant software version 1.5.3.17 (Max Planck Institute of Biochemistry in Martinsried, Germany) [24].

### Isolation of CAFs

CAFs and primary cancer cells were isolated from gastric tumor tissues by primary culture, while NFs were derived from the paired adjacent normal tissues [25]. The paired NFs and CAFs were further identified by the presence of CAF-specific markers (α-SMA, FAP, and FSP1).

### Determination of lipid ROS levels

To determine the levels of lipid-ROS, cells were seeded in 6-well plate, and the culture media was replaced by serum-free media containing 10 μmol/L 2′,7′-dichlorodihydrofluorescein diacetate (Sigma) and placed in dark for 30 min, gently shaken every 5 min. Cells were harvested by centrifuging at 1000 rpm for 5 min and washed 3 times with serum-free media followed by re-suspending in serum-free media, then incubated with 5 μl 7-aminoactinomycin D (KeyGEN Bio-TECH, Nanjing, China) in dark for 5 min. Fluorescence was determined at an excitation wavelength of 488 nm and an emission wavelength of 525 nm. The average intensity of fluorescence in each group indicated the amount of ROS within cells.

### Immunofluorescence

Cells were cultured on four-well chamber slides. At the time of harvest, cells were fixed with 4% paraformaldehyde and then permeabilized with 0.01% Triton X-100 for 10 min. Then cells were treated with anti-α-SMA antibody (Abcam, ab124964), anti-FAP antibody (Abcam, ab53066), and anti-FSP1 (Abcam, ab124805). In addition, all samples were treated with 40, 6-diamidino-2-phenylindole dye for nuclear staining (358 nm). For confocal microscopy, a Nikon C2 Plus confocal microscope was used.

### Determination of cell death

Cells death was determined by using PI (Roche) assay. Briefly, cells were seeded in a 12-well plate and treated with indicated drugs. Then cells were harvested and stained with 2 μg/ml PI. Dead cells (PI-positive cells) were analyzed using a BD Accuri C6 flow cytometer (BD Biosciences).

### Mitochondrial membrane potential (MMP)

Cells were seeded in a 6-well plate, and 0.5 mM TMRE was added and incubated for 30 min. Excess TMRE was removed by washing the cells with PBS. Labeled cells were trypsinized and resuspended in PBS plus 2% FBS. Fluorescence at Ex/Em = 549/575 nm was analyzed using a flow cytometer.

### RNA isolation and quantitative RT-PCR

Assays to quantify mature miRNAs were conducted as previously described with slight modifications [26, 27]. Total RNA was extracted from the cultured cells and tissues using TRIzol Reagent (Invitrogen) according to the manufacturer’s instructions. miR-522 determination was performed using Taqman microRNA probes (Catalog number: 4427975, ThermoFisher, US). All of the reactions were run in triplicate. After the reactions were complete, the cycle threshold (C_T_) data were determined using fixed threshold settings, and the mean C_T_ was determined from triplicate PCRs. A comparative C_T_ method was used to compare each condition to the control reactions. U6 snRNA was used as an internal control of miRNAs, and mRNA levels were normalized to GAPDH. The relative amount of gene normalized to control was calculated with the eq. 2^-ΔCT^, in which ΔC_T_ = C_T gene_-C_T_ control.

### The miRNA target prediction

The miRNA target prediction and analysis were performed with the algorithms from TargetScan (http://www.targetscan.org/), PicTar (http://pictar.mdc-berlin.de/) and miRanda (http://www.microrna.org/).

### Western blotting

The ALOX15, USP7 and hnRNPA1 expression were assessed by western blotting analysis and samples were normalized to GAPDH. Protein extraction was blocked with PBS-5% fat-free dried milk at room temperature for 1 h and incubated at 4 °C overnight with anti-ALOX15 (1:1000, Santa cruz), anti-hnRNPA1 (1:1000, Santa cruz), anti-CD63 (1:2000, Abcam), anti-TSG101 (1:1000, Santa Cruz), anti-Alix (1: 1000, Santa Cruz), anti-ubiquitin (1:1000, Santa Cruz), anti-α-SMA (1: 1000, Abcam), anti-FAP (1: 1000, Abcam), andti-FSP1 (1: 1000, Abcam), anti-CEA (1:1000, Abcam), anti-CK-18 (1:1000, Abcam) and anti-GAPDH (1:3000, Santa Cruz) antibodies respectively.

### Immuno-precipitation

Immuno-precipitation using anti-hnRNPA1 antibody was performed at 48 h or 72 h after treatment. Cells were lysated by the lysis buffer containing 150 mM KCl, 25 mM Tris-HCl, pH 7.4, 5 mM EDTA, 0.5% Triton X-100, 5 mM dithiothreitol (DTT), PMSF and cocktail. The supernatant was mixed with hnRNPA1 antibody (Abcam) overnight at 4 °C, and then co-cultured with beads (santa cruz) for 2–4 h at RT. The beads were washed five times in lysis buffer followed by western blotting (WB) analysis.

### Biotin miRNA pull-down assay

The cell lysates and exosomal lysates of CAFs were incubated overnight at 4 °C, with 100 pmol of synthetic single-stranded miR-522 or controlled miR-24 oligonucleotides containing a biotin modification. Agarose beads (Invitrogen, USA) were added to each binding reaction, which was further incubated at 4 °C for 4 h. Precipitates were washed five times and boiled in SDS buffer, followed by western blotting analysis or RT-qPCR analysis.

### Luciferase assay

The reporter plasmid p-MIR-ALOX15 containing the predicted miR-522 targeting regions was designed by Genescript (Nanjing, China). Part of the wild-type and mutated 3′-UTR of ALOX15 was cloned immediately downstream of the firefly luciferase reporter. The 2 mg of beta-galactosidase expression vector (Ambion) was used as a transfection control. For the subsequent luciferase reporter assays, 2 mg of firefly luciferase reporter plasmid, 2 mg of beta-galactosidase vector and equal doses (200 pmol) of mimics, inhibitors or scrambled negative control RNA were transfected into the prepared cells. At 24 h after transfection, cells were analyzed using the Dual Luciferase Assay Kit (Promega) according to the manufacturer’s instructions. Each sample was prepared in triplicate and the entire experiment was repeated three times.

### Immunohistochemistry (IHC)

The tumors were fixed in 4% paraformaldehyde, embedded in paraffin, sectioned and then stained with corresponding antibodies (Abcam). Quantitative analysis was conducted by quantifying the fluorescence intensity from at least five sections.

### Hematoxylin-eosin (HE) staining

Tissues were fixed in 10% formalin, processed and embedded in paraffin. Multiple sections (10 μm in thickness) were prepared and stained with haematoxylin and eosin for morphological observation.

### Establishment of tumor in nude mice

Equal number of SGC7901 cells and primary CAFs isolated from GC tissues were injected into nude mice by orthotopic implantation. Briefly, 5 × 10^5^ SGC7901cells and CAFs were injected subcutaneously for one mouse. These tumor-implanted mice were injected with either cisplatin (5 μg/g) or saline every 5 days since the day ten, and were sacrificed and tumors were removed at the 30th Day.

### Statistics

All experiments were performed in triplicate, and the results are presented as the mean value ± standard deviation. The data were statistically analyzed using Student’s t-test in SPSS statistical software, with *p* < 0.05 considered statistically significant. * indicates *p* < 0.05; ** indicates *p* < 0.01 and *** indicates *p* < 0.001.

## Results

### Screening of the key genes related to ferroptosis in GC

**Fig. 1:**
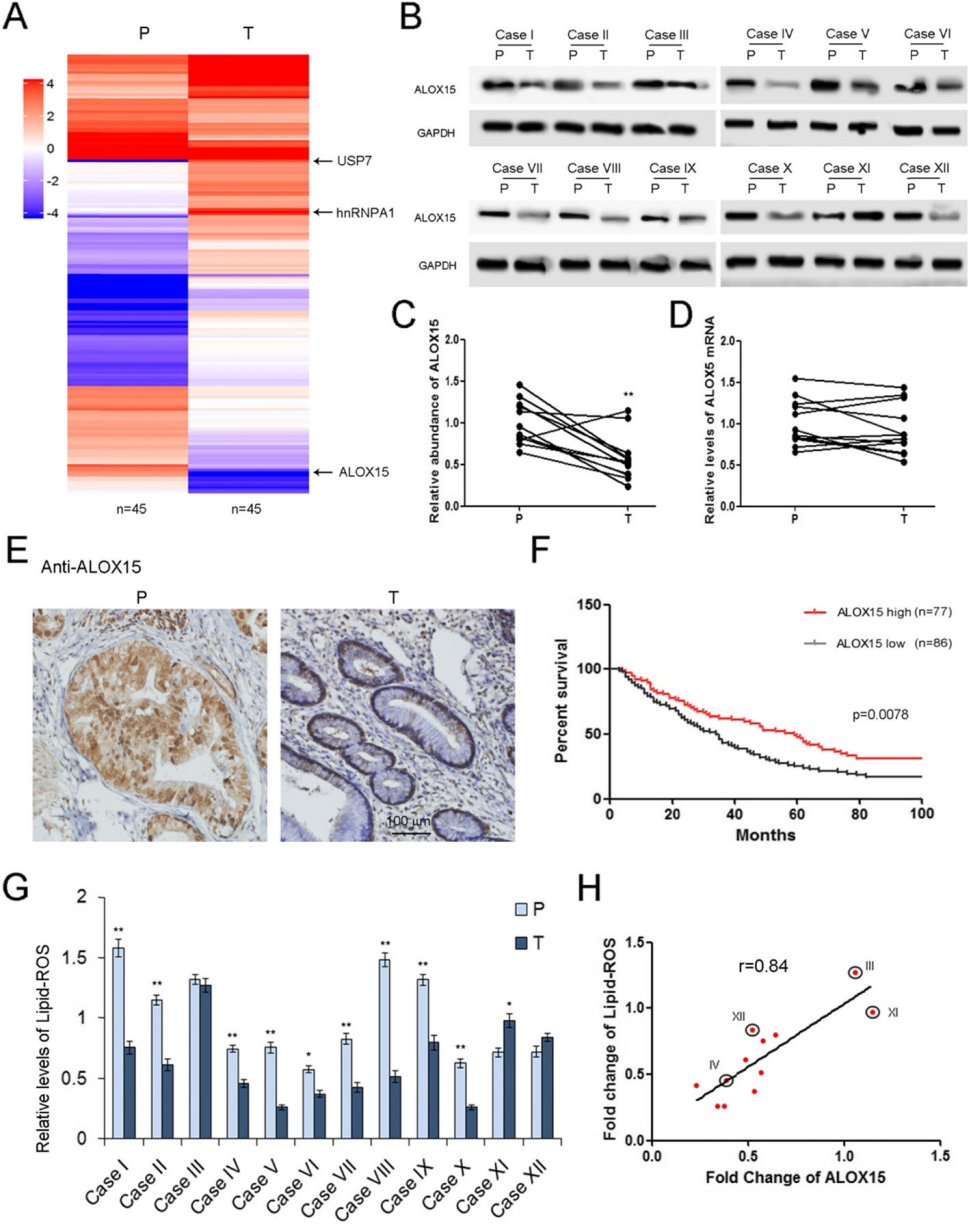
ALOX15 is related to ferroptosis in gastric cancer. **a**. Mass spectrum analysis of gene expression changes in gastric tumor tissues (*n* = 45). The heatmap depicts the relative protein abundance in tumor tissues (T) and paired para-carcinoma tissues (P). **b**. Validation of ALOX15 dys-regulation in GC by using western blotting analysis (*n* = 12). **c**. Quantitative analysis of (**b**) (n = 12). **d**. Relative levels of ALOX15 mRNA in gastric tumor tissues (n = 12). **e**. Analysis of ALOX15 distribution in tumor tissues by using IHC (n = 12). **f**. High expression of ALOX15 predicts poor overall survival in GC. Patients were divided into ALOX15 high group (*n* = 77) and ALOX15 low group (*n* = 86) base on the average value of ALOX15 protein levels. **g**. Relative levels of lipid-ROS levels in gastric tumor tissues (n = 12). **h**. ALOX15 is positively co-related with Lipid-ROS production (n = 12). ** indicates *p* < 0.01

Previous studies have shown that 15-lipoxygenase (ALOX15) plays a key role in mediating the production of Lipid ROS in various types of tissues and tumors [28–30]. We firstly compare the GC specific proteins by using Mass Spectrum, and a panel of proteins was significantly dys-regulated in GC tumor tissues (T) compared with para-carcinoma tissues (P). ALOX15, one of the key genes leading to ferroptosis, showed a sharp decrease, while the levels of USP7 and hnRNPA1 were clearly increased in tumor tissues (Fig. 1a). Subsequently, ALOX15 protein was measured by WB analysis and ALOX15 was determined by RT-qPCR. The expression pattern of ALOX15 was validated in 12 gastric cancer patients, and ALXO15 protein showed an overall downward trend, but ALOX15 mRNA had no evident changes (Fig. 1b, c and d). Therefore, ALOX15 is mainly regulated in GC cells at the post-transcriptional level. The HE staining was performed to confirm the morphological features of tumor tissues and para-carcinoma tissues (Supplemental Figure 1A). We also checked the distribution of ALOX15 by using IHC assay, and ALOX15 was mostly expressed in gland cells of para-carcinoma tissues, and was expressed in small amounts in adenoma cells (Fig. 1e). The expression patterns of USP7 (Supplemental Figure 2A), hnRNPA1 (Supplemental Figure 2B), ALOX15 (Supplemental Figure 2C) in individuals were also displayed to show visualizing differences. GC patients were divided into ALOX15 high group (*n* = 77) and ALOX15 low group (*n* = 86) base on the mean value of ALOX15 protein levels. High levels of ALOX15 protein are also associated with better overall survival (OS) of gastric cancer patients (Fig. 1f), suggesting that ALOX15 acts as an anti-cancer factor in GC. Since Lipid-ROS is an important metabolite generated by ALOX15 [31], the level of Lipid-ROS was clearly decreased in tumor tissues (Fig. 1g), and was positively related with ALOX15 (Fig. 1h). These data implied that the ALOX15 plays a key role in mediating Lipid-ROS production in gastric tumors.

### Exosomal miR-522 serves as a potential up-stream regulator of ALOX15

Serum exosomes of both normal subjects and GC patients were isolated by ultra-centrifugation and were observed by using electron microscope (Supplemental Figure 3A). Exosome markers were detected by western blotting assay, and CD63, Tsg101 and Alix were contained in these exosomes (Supplemental Figure 3B). Nano-tracking analysis showed that the diameters of vesicles isolated from serum were chiefly 30–150 nm, which is a typical size of exosomes (Supplemental Figure 3C). Since no clear relevance was observed between ALOX15 protein and ALOX15 mRNA, we inferred that ALOX15 was regulated by miRNAs at the post-transcriptional level. miR-522 was predicted to interact with ALOX15 mRNA, and the binding region was shown in Supplemental Figure 3D. It was reported that miR-522 promotes tumor progression in non-small cell lung cancer, colorectal cancer and hepatocellular carcinoma [32–34], but the role of miR-522 in gastric cancer has not been well defined yet. Our data showed that miR-522 was up-regulated in both serum exosomes and tumor tissues of GC patients, and exo-miR-522 positively correlated with tumor grade (Supplemental Figure 3E and 3F). Moreover, miR-522 is found to be negatively linked with ALOX5 expression (Supplemental Figure 3G), as well as Lipid-ROS (Supplemental Figure 3H). GC patients were divided into miR-522 high group (*n* = 184) and miR-522 low group (*n* = 162) base on the average value of miR-522. The survivorship curve was generated from the online database (Kaplan-Meier Plotter, https://kmplot.com/analysis/), and high levels of miR-522 predict poor survival in GC (Supplemental Figure 3I). Therefore, these results suggested the potential clinical relevance between miR-522, ALOX15 and lipid-ROS accumulation.

### Exosomal miR-522 mainly derived from CAFs in tumor microenvironment

**Fig. 2:**
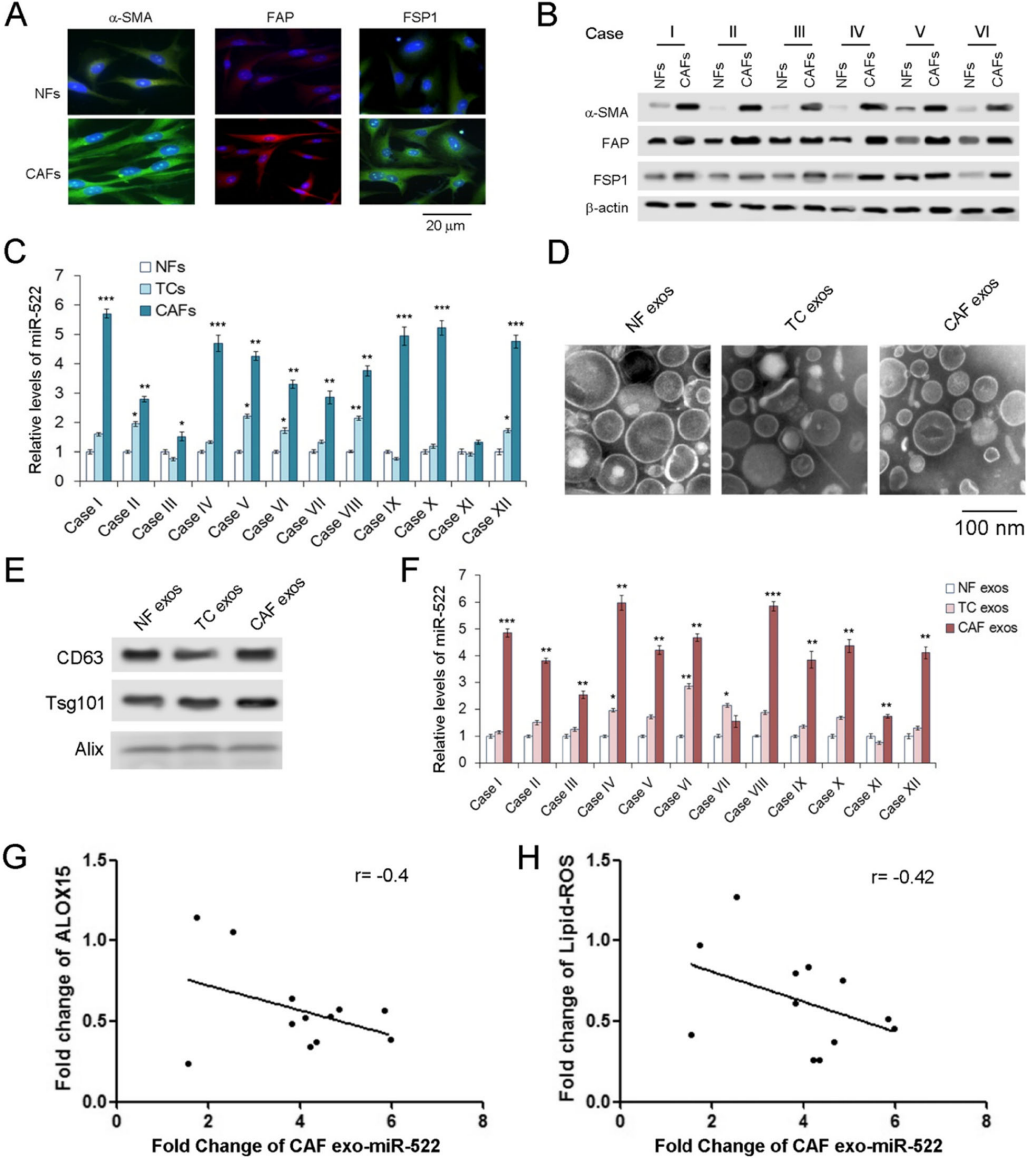
Ferroptosis-related exo-miR-522 mainly derived from CAFs. **a**. Immunofluorescence staining for α-SMA, FAP, and FSP1 expression of NFs and CAFs (scale bar, 20 μm). **b**. Western blot analysis of α-SMA, FAP, and FSP1 protein levels in six paired NFs and CAFs (*n* = 6). **c**. Comparison of miR-522 levels in primary NFs, tumor cells (TCs) and CAFs (n = 12). **d**. TEM image of exosomes isolated from primary NFs, TCs and CAFs (scale bar, 100 nm). **e**. Western blotting analysis of CD63, Alix, and Tsg101 in exosomes. **f**. Relative levels of miR-522 in the exosomes described above (n = 12). **g**. The levels of exo-miR-522 derived from CAFs is negatively linked with ALOX15 expression (n = 12). **h**. CAF exo-miR-522 is negatively related with lipid-ROS levels (n = 12). ** indicates *p* < 0.01 and *** indicates *p* < 0.001

Although we have proved that exosomal miR-522 was clearly up-regulated in GC, the origin of miR-522 remains unclear. Tumor microenvironment is composed of various cell types, and CAFs contribute the largest proportion of stromal cells [11, 35, 36]. CAFs in tumor tissues and normal fibroblasts (NFs) in para-carcinoma tissues were isolated, and levels of miR-522 were measured in both primary cells and exosomes. All the markers of CAFs, α-SMA, FAP and FSP1 are obviously increased in CAFs compared with NFs (Fig. 2a and b). The markers for GC cells, CEA and CK-18, were not expressed in NFs and CAFs, and tumor cells (TCs) do not contain α-SMA, FAP and FSP1 (Supplemental Figure 4A). The highest concentration of miR-522 was found in CAFs, compared with tumor cells and NFs (Fig. 2c). Exosomes of NFs, TCs and CAFs were isolated and photographed as described above (Fig. 2d), and the markers of exosomes were detected by western blotting analysis (Fig. 2e). The level of miR-522 ranked top in CAF exosomes, followed by TC exosomes, and the content of miR-522 in CAF exosomes was absolutely dominant among the three types of exosomes (Fig. 2f). As is expected, exosome miR-522 derived from CAFs also showed a negative relationship with ALOX15 and Lipid-ROS (Fig. 2g and h). These data demonstrated that exo-miR-522 in GC tumor microenvironment are mainly secreted by CAFs.

### CAFs secreted exo-miR-522 suppresses ferroptosis of GC cells

**Fig. 3:**
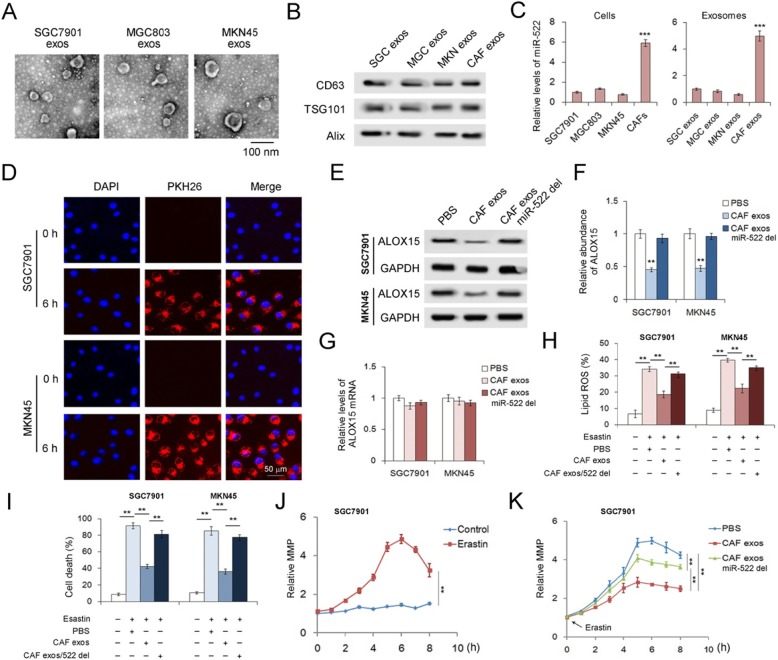
CAFs secrete exo-miR-522 to suppress ferroptosis of GC cells. **a**. TEM images of exosomes isolated from primary NFs, TCs and CAFs (scale bar, 100 nm). **b**. WB analysis of exosomal markers in the exosomes isolated from GC cell lines and CAFs. **c**. Quantification of miR-522 in both cells and exosomes (*n* = 3). **d**. Confocal microscopy image of the internalization of fluorescently labeled CAF exosomes in SGC7901 cells and MKN45 cells. Scale bars, 50 μm. **e**. Effects of CAF-secreted miR-522 on the expression of ALOX15 in GC cells (n = 3). **f**. Quantitative analysis of (**e**) (n = 3). **g**. Relative levels of ALOX15 mRNAs in GC cells treated as described above (n = 3). **h**-**k**. Exo-miR-522 derived from CAFs suppresses erastin-induced ferroptosis in GC cells. CAF exo-miR-522 inhibits lipid-ROS accumulation (**h**), decreases erastin-induced cell death (**i**) and reduces abnormal increase of MMP (**j**, **k**). ** indicates *p* < 0.01 and *** indicates *p* < 0.001

To test the function of exo-miR-522 derived from CAFs in the regulation of ferroptosis of cancer cells, CAF exosomes were isolated and co-cultured with human gastric cancer cell lines. Photos of exosomes secreted by SGC7901 cells, MGC803 cells and MKN45 cells were shown in Fig. 3a, and these exosomes also contained CD63, TSG101 and Alix (Fig. 3b). The amount of miR-522 was 6 times in CAFs than in GC cell lines, and the same trend was observed in exosomes (Fig. 3c). CAF exosomes were co-cultured with GC cells, and PKH-26 labeled CAF exosomes were detected in both SGC7901 cells and MKN45 cells at 6 h (Fig. 3d), indicating that exosomes derived from CAFs can fuse with GC cells efficiently. And CAFs exosomes dramatically inhibited ALOX15 expression in GC cells without affecting the content of ALOX15 mRNA, which is dependent on miR-522 (Fig. 3e, f and g). Furthermore, exo-miR-522 was proved to effectively suppress erastin-induced Lipid-ROS accumulation (Fig. 3h) and ferroptosis (Fig. 3i) in GC cells. SGC7901 cells treated with erastin showed a clear increase of mitochondrial membrane potential (MMP) (Fig. 3j), and CAF exosomes partly reversed the damage of erastin to mitochondria (Fig. 3k). To conclude, exo-miR-522 secreted by CAFs suppresses ALOX15 expression and down-regulate the level of ferroptosis in GC cells.

### Validation of the direct interaction between miR-522 and ALOX15

Three miRNA, miR-522, miR-106a and miR-125b were predicted as the upstream regulators of ALOX15 (Supplemental Figure 5A), however, miR-106a and miR-125b showed little difference between CAFs and NFs (Supplemental Figure 5B and 5C). Thus, miR-522 was selected as the potential regulator of ALOX15, and the predicted binding sites between miR-522 and ALOX15 mRNA were shown in Supplemental Figure 6A. Subsequently, we performed the luciferase reporter assay to further reveal the direct contact of miR-522 and ALOX15 mRNA. A luciferase reporter plasmid containing miR-522-binding region was constructed, and the plasmids containing the reversed sequences of the miR-522-binding sites (mut) were used as negative control. Either the transfection of miR-522 mimics or treatment of CAF exosomes lead to a significant suppression of luciferase activity; while the knock-down of miR-522 relatively increased the level of luciferase (Supplemental Figure 6B). Overexpressed miR-522 dramatically decreased ALOX15 levels, while transfection of miR-522 inhibitors promoted ALOX15 expression in both SGC7901 and MKN45 cells (Supplemental Figure 6C and D). But the mRNA of ALOX15 showed little change under the treatment of miR-522 (Supplemental Figure 6E), suggesting that miR-522 negatively regulates ALOX15 expression at the post-transcriptional level. Immuno-precipitation using biotin labeled miR-522 was performed in SGC7901 cells, and biotin-miR-24 was used as a negative control. ALOX15 mRNA was only detected in the biotin labeled miR-522 group (Supplemental Figure 6F).

As is expected, overexpression of miR-522 showed the same effects as CAF exosomes on blocking the accumulation of lipid-ROS (Supplemental Figure 6G), suppressing erastin-induced cell death (Supplemental Figure 6H), and reversing erastin-induced MMP increase (Supplemental Figure 6I). And the knockdown of miR-522 by inhibitors showed a completely opposite function (Supplemental Figure 6G-I). To investigate whether miR-522 is involved in the other types of cell death, we treated GC cells with apoptosis activator (stauroporine) and inhibitor (ZVAD-FMK), and necrosis activator (TNF-α) and inhibitor (necrosulfonamid), respectively. Both stauroporine and TNF-α lead to significant increase of cell death in GC cells, however, miR-522 failed to reduce the ratio of cell death (Supplemental Figure 6 J and 6 K). Thus, miR-522 efficiently restrained lipid-ROS production and ferroptosis by directly targeting ALOX15 in GC cells.

### USP7 promotes miR-522 secretion by stabilizing hnRNPA1

**Fig. 4:**
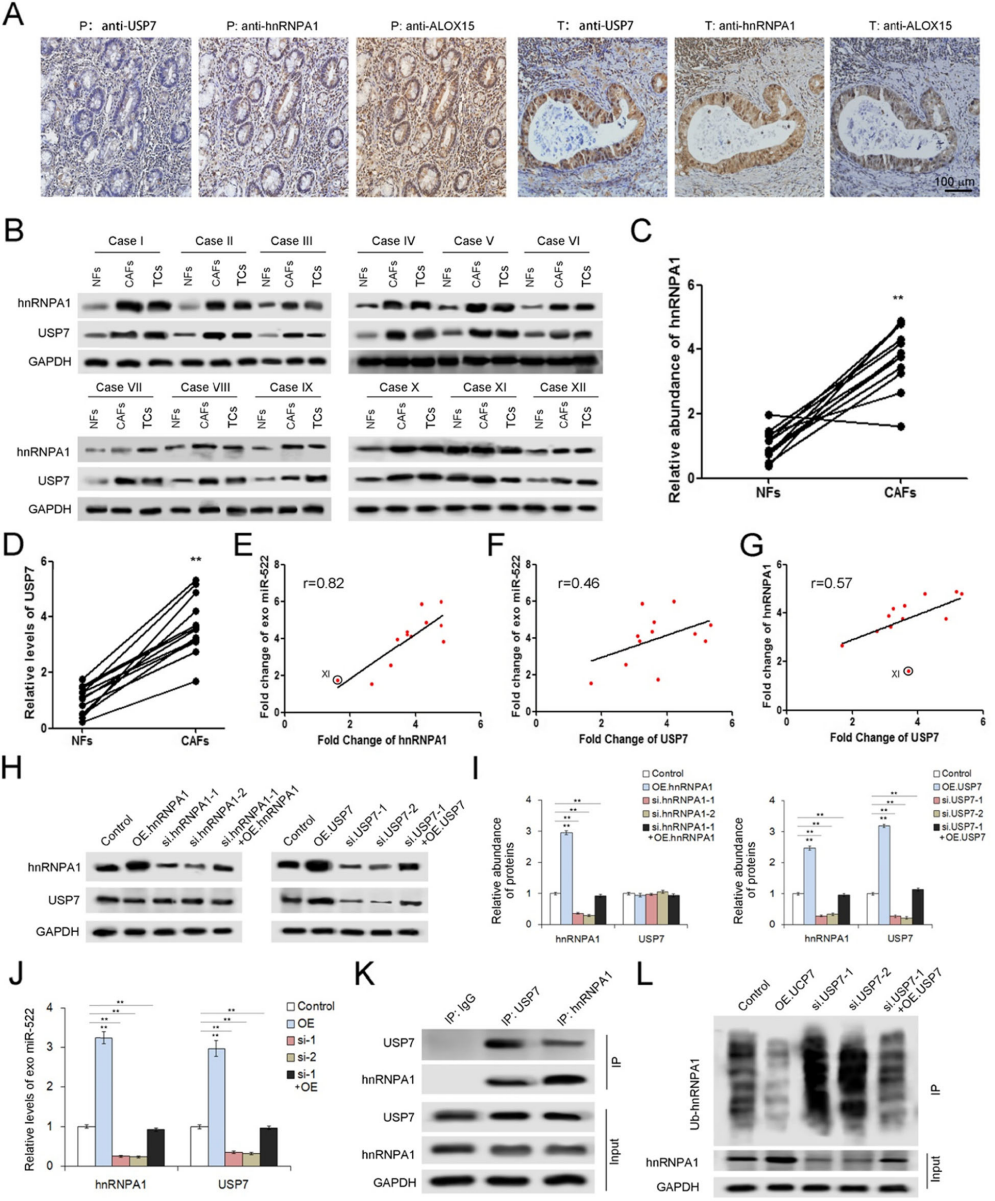
USP7 promotes miR-522 secretion by regulating deubiquitination of hnRNPA1 in CAFs. **a**. IHC analysis of USP7, hnRNPA1 and ALOX15 by using serial sections in paired para-carcinoma tissues and tumor tissues (n = 12). **b**. WB analysis of hnRNPA1 and USP7 in 12 paired CAFs and NFs (n = 12). **c**-**d**. Quantitative analysis of hnRNPA1 (**c**) and USP7 (**d**) described in (**b**) (n = 12). **e**-**g**. Analysis of the correlation between USP7, hnRNPA1 and exo-miR-522 in CAFs. HnRNPA1 is positively related with exo-miR-522 (**e**), and USP7 also showed positive relevance with both exo-miR-522 (**f**) and hnRNPA1 (**g**) (n = 12). **h**. WB analysis of hnRNPA1 and USP7 in CAFs treated with hnRNPA1 overexpression plasmids (OE.hnRNPA1), hnRNPA1 siRNA (si.hnRNPA1), USP7 overexpression plasmids (OE.USP7) and USP7 siRNA (si. USP7) respectively (n = 3). **i**. Quantitative analysis of (**g**) (n = 12). **j**. USP7/hnRNPA1 promotes miR-522 secretion (n = 3). **k**. Immuno-precipitation shows the direct interaction between USP7 and hnRNPA1 in CAFs (n = 3). **l**. WB analysis of hnRNPA1 ubiquitination in CAFs treated with OE.USP7 or si. USP7 (n = 3). ** indicates *p* < 0.01

It has been reported that hnRNP family is required for the packaging into exosomes of a list of mRNAs and non-coding RNAs [17, 37, 38]. In the current study, hnRNPA1 has been screened out by MS for its up-regulation in gastric tumor tissues. IHC analysis using serial sections showed the correlations between USP7, hnRNPA1 and ALOX15 (Fig. 4a). We next checked the expression of hnRNPA1 as well as USP7 in primary CAFs isolated from the 12 tumor tissues described above, and it is shown that hnRNPA1 and USP7 are highly expressed in CAFs compared with paired NFs (Fig. 4b, c and d). Moreover, the levels of both hnRNPA1 and USP7 are positively related to exosomal miR-522 (Fig. 4e and f), implying that USP7 and hnRNPA1 are involved in miR-522 secretion from CAFs. Interestingly, deubiquitination-related USP7 seems to play an important role in increasing hnRNPA1 levels (Fig. 4g).

To further investigate the inner link between USP7, hnRNPA1 and miR-522, the plasmids containing either hnRNPA1 or USP7 coding sequences, as well as the siRNAs of both genes were constructed. Transfection of plasmids markedly up-regulated both hnRNPA1 and USP7 expression, while the application of siRNAs lead to the dramatical reduction of both genes (Fig. 4h and i). The overexpression of either hnRNPA1 or USP7 in CAFs promotes the packaging of miR-522 into exosomes; however, the knockdown of both genes relatively decreased the levels of exo-miR-522 (Fig. 4j). The rescue experiments showed that overexpressed hnRNPA1 or USP7 partly neutralized the effects of respective siRNAs on exo-miR-522 (Fig. 4h-j). Next, USP7 can be detected in the product of immuno-precipitation assay by using the anti-hnRNPA1 antibody, and vice versa (Fig. 4k). In addition, USP7 also showed a negative link with the levels of hnRNA1 ubiquitination (Fig. 4l). Collectively, these results indicated that USP7 stabilizes hnRNPA1 in CAFs via deubiquitination, resulting in the enhanced secretion of exosomal miR-522.

### hnRNPA1 directly mediates miR-522 packaging into CAF-derived exosomes

**Fig. 5:**
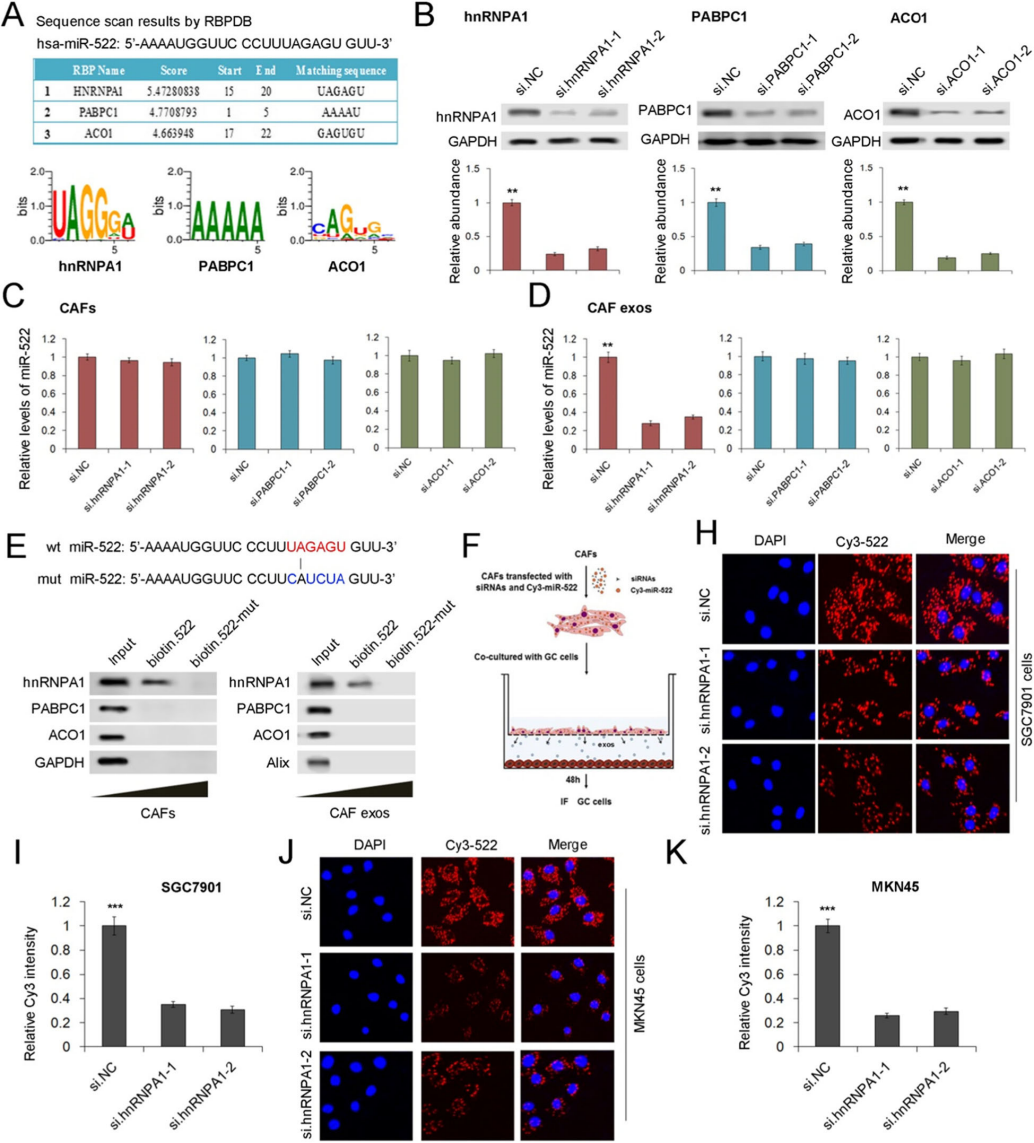
Direct evidence for hnRNPA1 mediated miR-522 packaging into CAF exosomes. **a**. RBPDB analysis of the specific interaction between miR-522 and RBP motifs (threshold 0.5). **b**. WB analysis of hnRNPA1, PABPC1 and ACO1 expression in CAFs transfected with corresponding siRNAs (n = 3). **c**. Relative levels of miR-522 in CAFs transfected with siRNAs (n = 3). **d**. Quantification of miR-522 in CAF exosomes treated as described above (n = 3). **e**. Detection of hnRNPA1 protein in the samples derived from miR-522 pull downs performed in CAFs and CAF exosomes (n = 3). **f**. Schematic diagram for the cell co-culture model of CAFs and SGC7901 cells. **h**. Capture of CAF-exosome delivered Cys-miR-522 by SGC7901 cells co-cultured with CAFs (n = 3). **i**. Quantitative analysis of (**h**) (n = 3). **j**. Capture of CAF-exosome delivered Cys-miR-522 by MKN45 cells co-cultured with CAFs (n = 3). **k**. Quantitative analysis of (J) (n = 3). ** indicates *p* < 0.01; *** indicates *p* < 0.001

To further validate the role of hnRNPA1 in selectively packaging miR-522 into exosomes, the potential miR-522 binding proteins were predicted by using RBPDB (the database of RBP specificities, http://rbpdb.ccbr.utoronto.ca/). The top three RNA-binding proteins were shown in Fig. 5a (threshold 0.5), and hnRNPA1 has the highest score. We also designed siRNAs for PABPC1 and ACO1, and both PABPC1 and ACO1 were obviously knocked down by siRNAs (Fig. 5b). The transfection of these siRNAs showed little effects on the expression miR-522 in CAFs (Fig. 5c), but the down-regulation of hnRNPA1, instead of PABPC1 and ACO1, significantly decreased miR-522 levels in CAF exosomes (Fig. 5d). Wild type and mutated miR-522 were labeled with biotin and were transfected into CAFs, followed by immuno-precipitation. It was shown that only hnRNPA1was detected in the production of co-IP using wild type biotin-miR-522 (Fig. 5e). Primary CAFs and SGC7901 cells were co-cultured by using the 0.4 μm polyester membrane (Fig. 5f). Briefly, Cy3 labeled miR-522 was transfected into CAFs, and exosomes derived from CAFs passed though the membrane to fuse with lower GC cells. Cys-miR-522 can be clearly detected in both SGC7901 cells and MKN45 cells, and silencing of hnRNPA1 in CAFs blocked cys-miR-522 transfer from CAFs to GC cells (Fig. 5h-k). Therefore, these data provide direct evidence to show that hnRNPA1 play a vital role in mediating the packaging of miR-522 into CAF exosomes.

### Chemo-toxicity promotes miR-522 secretion from CAFs by activating USP7/hnRNPA1 pathway

**Fig. 6:**
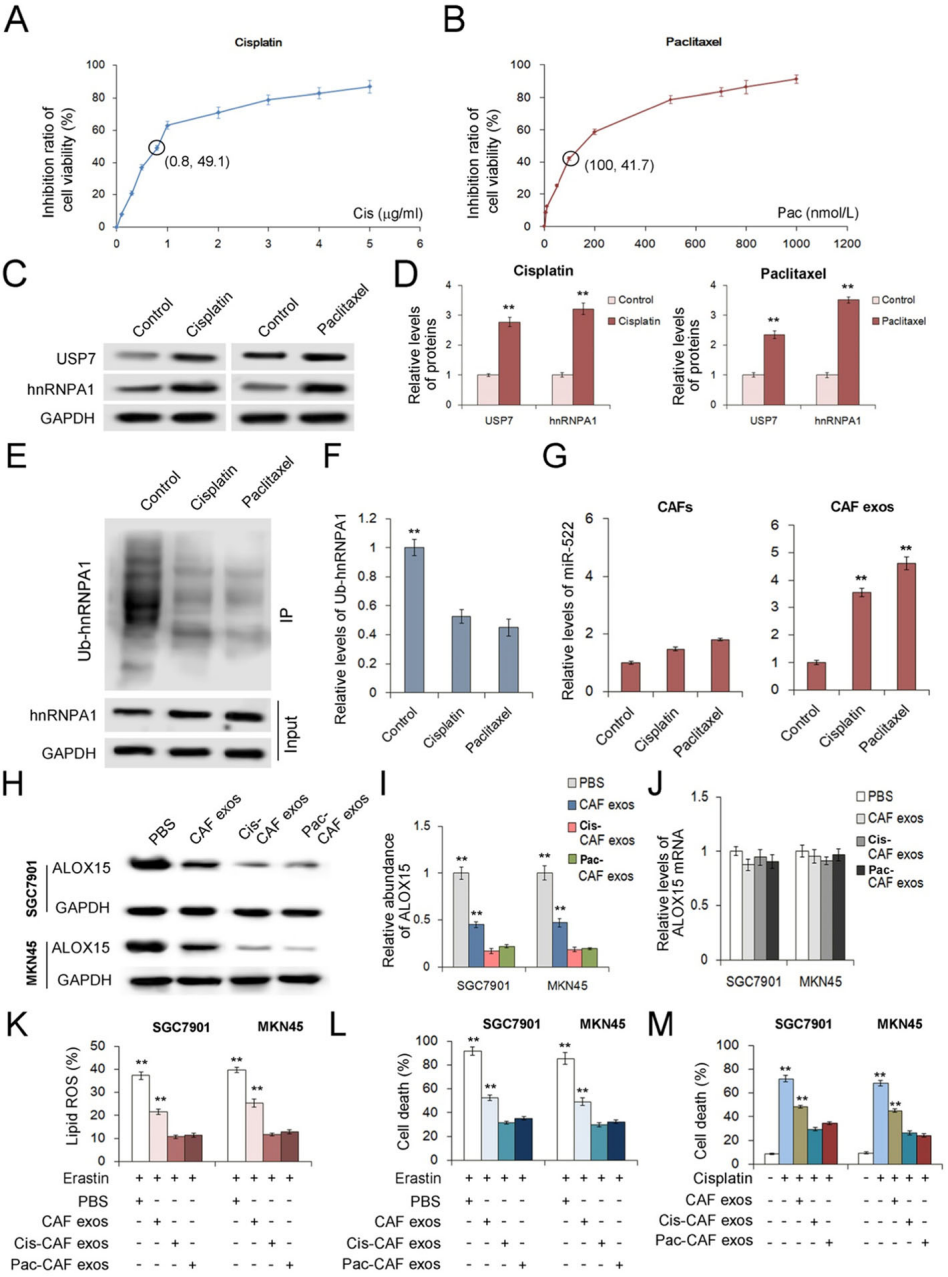
Chemo-toxicity up-regulates USP7/hnRNPA1 and promotes miR-522 secretion from CAFs. **a**-**b** The effects of cisplatin (**a**) and paclitaxel (**b**) on cell viability of CAFs (n = 3). **c**. The expression of USP7 and hnRNPA1 in CAFs treated with sublethal doses of cisplatin (0.8 μg/ml) and paclitaxel (100 nmol/L) (n = 3). **d**. Quantitative analysis of (**c**) (n = 3). **e**. Chemo-toxicity reduces ubiquitination of hnRNPA1 (n = 3). **f**. Quantitative analysis of (**e**) (n = 3). **g**. Effects of cisplatin and paclitaxel on the expression and secretion of miR-522 (n = 3). **h**-**i**. Effects of exosomes derived from chemo-toxicity treated CAFs on ALOX15 expression in GC cells. The ALOX15 protein was determined by WB assay (**h**) and was quantified by gray analysis (**i**) (n = 3), and ALOX15 mRNA was checked by qRT-PCR (**j**) (n = 3). **k**. Exosomes of chemo-toxicity treated CAFs inhibit erastin induced lipid-ROS production in GC cells (n = 3). **l**. Exosomes of chemo-toxicity treated CAFs inhibit erastin induced cell death of GC cells (n = 3). **m**. Exosomes of chemo-toxicity treated CAFs contribute to acquired chemo-resistance of GC cells (n = 3). * indicates *p* < 0.05 and ** indicates *p* < 0.01

To access for the chemotherapy-induced damage responses in CAFs, we examined GC cells treated with sublethal doses of cisplatin or paclitaxel. The inhibitory effects of cisplatin and paclitaxel on cell viability of CAFs were shown in Fig. 6a and b, and 0.8 μg/ml cisplatin and 100 nmol/L paclitaxel were selected as the sublethal doses for CAFs respectively. It is clearly shown that either cisplatin or paclitaxel promoted USP7 and hnRNPA1 expression in CAFs (Fig. 6c and d); and chemo-toxicity also enhanced deubiquitination of hnRNPA1 (Fig. 6e and f), which lead to the up-regulation of miR-522 in exosomes without obviously change miR-522 expression in primary CAFs (Fig. 6g). This result suggested that chemotherapy-induced damage promoted miR-522 packaging into exosomes by increasing the expression of USP7 and reducing ubiquitination of hnRNPA1.

Exosomes isolated from CAFs treated with cisplatin (Cis-CAF exos) or paclitaxel (Pac-CAF exos) were co-cultured with GC cells, and they showed an even greater ability to inhibit ALOX15 protein expression than the control CAF exosomes without affecting ALOX15 transcription (Fig. 6h, i and j). Both Cis-CAF exos and Pac-CAF exos more efficiently reduced lipid-ROS production (Fig. 6k) and suppressed erastin-induced ferroptosis (Fig. 6l) in SGC7901 cells and MKN45 cells. Addition of cisplatin in the medium immensely caused high death ratio of GC cells, which was decreased by Cis-CAF exos and Pac-CAF exos (Fig. 6m).

These in vitro experiments provide evidence for that CAF exosomes contributes to decreased drug sensiticity during chemotherapy.

### In vivo role of USP7/hnRNPA1/miR-522 in regulating gastric tumor growth and chemo-sensitivity

**Fig. 7:**
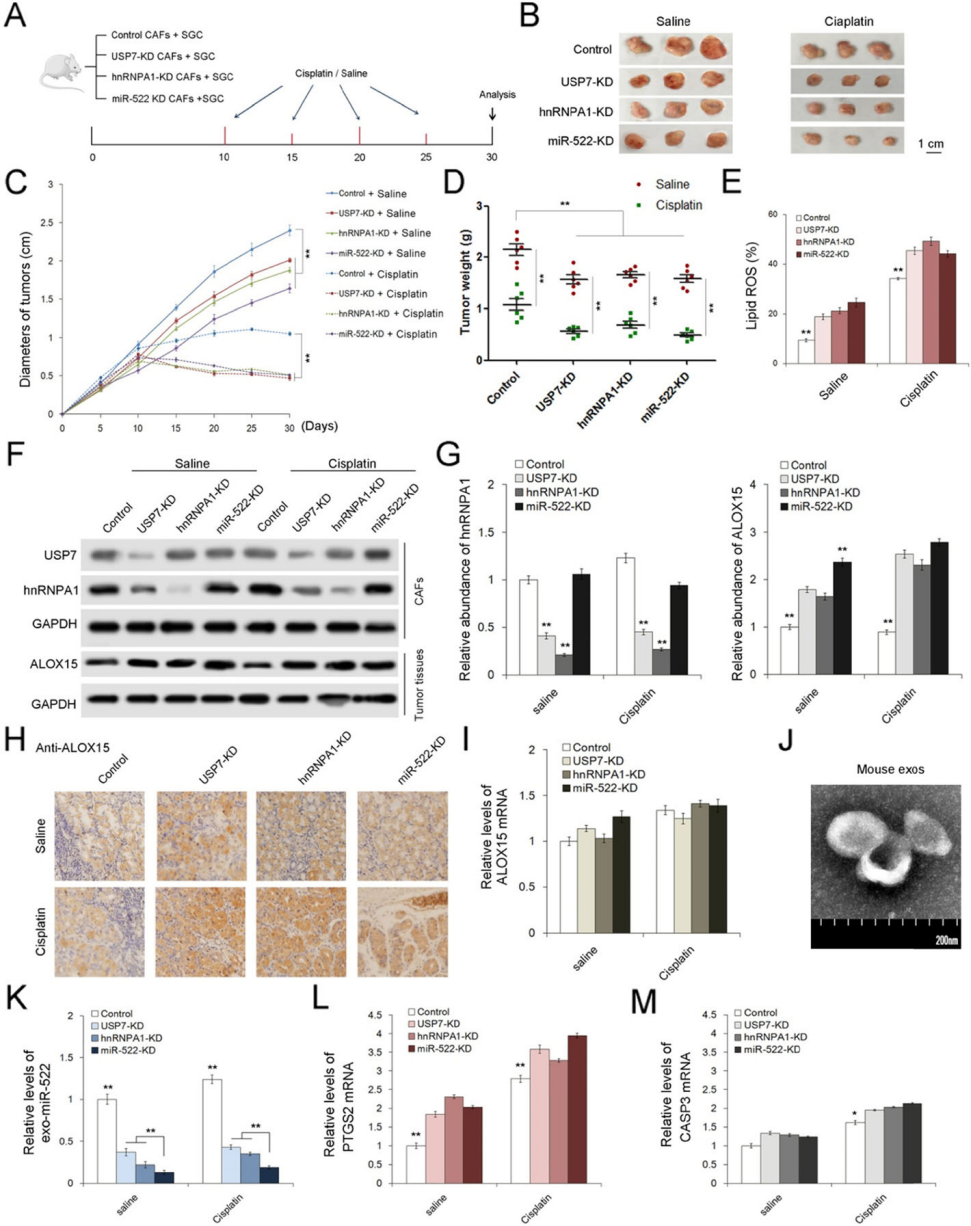
In vivo role of USP7/hnRNPA1/exo-miR-522 pathway in regulating ferroptosis and chemo-sensitivity of gastric tumors. **a**. Schematic description of the experimental design used to establish the animal model. **b**. Images of tumors in each group (*n* = 6). **c**. Alterations of tumor diameters in each group (n = 6). **d**. Weight measurements of the tumors described above (n = 6). **e**. Relative levels of lipid-ROS in tumors (n = 6). **f**. WB analysis of USP7 and hnRNPA1 in primary CAFs and ALOX15 in tumor tissues (n = 6). **g**. Quantitative analysis of (**f**). **h**. IHC analysis of ALOX15 distribution in tumor tissues (n = 6). **i**. Relative levels of ALOX15 in tumor tissues (n = 6). **j**. TEM images of exosomes isolated from mouse serum. **k**. Relative levels of miR-522 in serum exosomes (n = 6). **l**-**m**. Quantification of ferroptosis marker (**l**) and apoptosis marker (**m**) (n = 6). ** indicates *p* < 0.01

Finally, the role of USP7/hnRNPA1/miR-522 axis in affecting tumor growth and chemotherapeutic efficacy were evaluated in vivo. We generated three gastric fibroblast cell strains with the knockdown of USP7, hnRNPA1 and miR-522 respectively by using lenti-viruses containing shRNAs, and these fibroblast cell strains were mixed with SGC7901 cells for subcutaneous tumor implantation in mice (Fig. 7a). These tumor-bearing mice were then injected with either cisplatin (5 μg/g) or saline every 5 days since the day ten, and tumors were harvested at the 30th day. The knockdown of USP7, hnRNPA1 or miR-522 in CAFs observably suppressed tumor growth and enhanced sensitivity to cisplatin (Fig. 7b, c and d), but up-regulated Lipid ROS levels in tumors (Fig. 7e). We also collected primary CAFs isolated from mouse tumors, and it is clearly shown that knockdown of USP7 decreased hnRNPA1 protein levels; and the suppression of each of the three genes in CAFs lead to ALOX15 up-regulation in cancer cells (Fig. 7f, g and h). IHC assay by using anti-α-SMA antibody was performed to show that lenti-virus transduction did not influence the proliferation of CAFs in vivo (Supplemental Figure 7A). Treatment of cisplatin relatively promoted the expression of USP7/hnRNPA1 in CAFs, as well as ALOX15 in cancer cells (Fig. 7f, g and h), but the mRNA of ALOX15 remained little changed (Fig. 7i). Plasma exosomes were isolated from these mice (Fig. 7j), and levels of exosomal miR-522 showed a sharp decrease in the USP7-KD and hnRNPA1-KD groups, and were lowest in the miR-522-KD group (Fig. 7k). The ferroptosis marker, PYGS2, was obviously up-regulated, while CASP3, which is an apoptosis marker, showed on slight increase with the knockdown of USP7, hnRNPA1 and miR-522 in CAFs (Fig. 7l-m).

Taken together, we provided in vivo evidence to demonstrate that USP7/hnRNPA1 promotes exo-miR-522 secretion from CAFs, decreasing lipid-ROS levels in tumors and facilitating tumor growth. In addition, these data also illustrated that effects of chemotherapeutic drugs can be improved via blocking miR-522 secretion.

### Effects of USP7/hnRNPA1/exo-miR-522 pathway on the survival of tumor-implanted mice

**Fig. 8:**
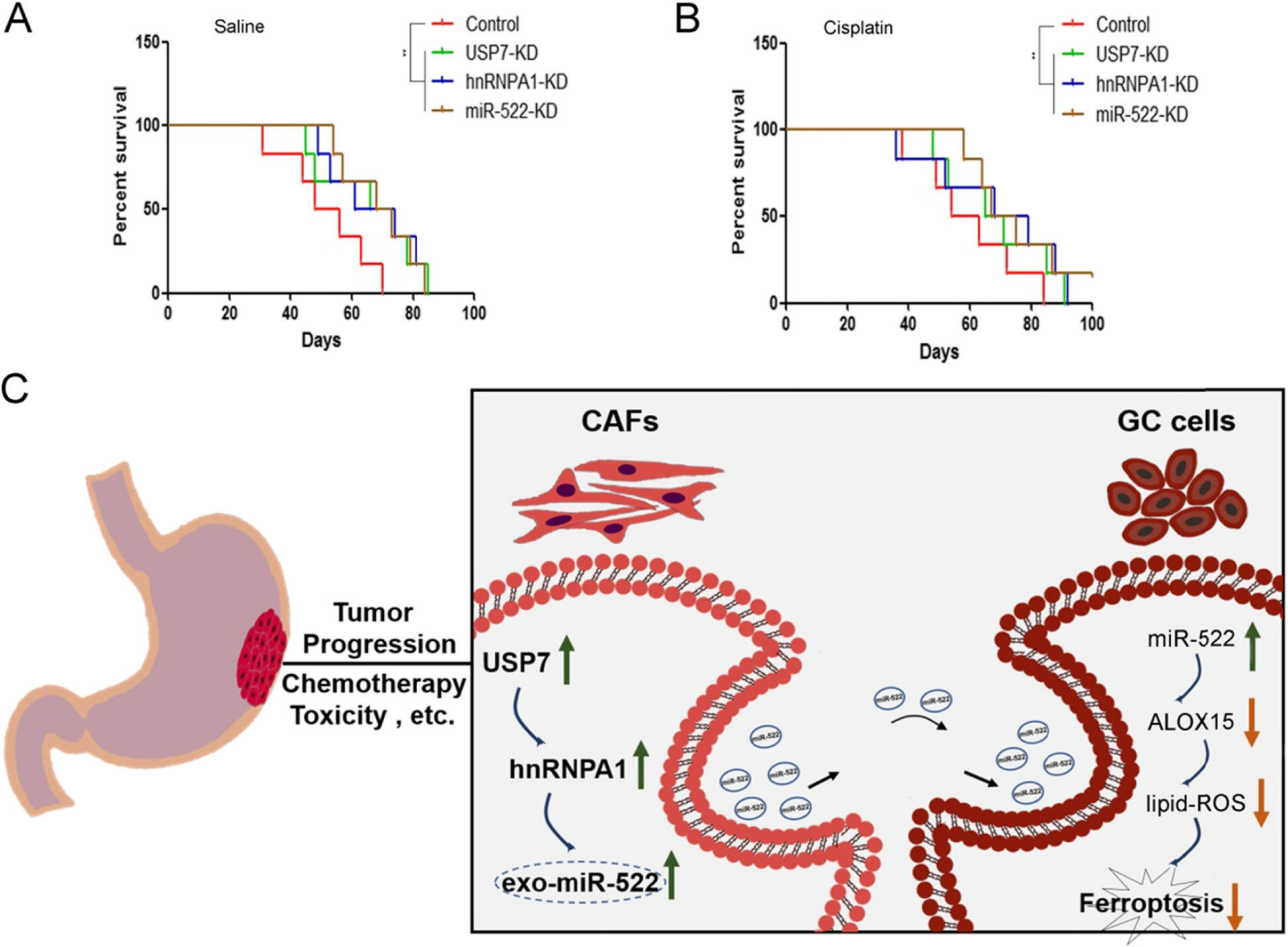
Effects of USP7/hnRNPA1/exo-miR-522 pathway on the survival of tumor-implanted mice. **a**-**b**. Kaplan–Meier curves of mice in the saline groups (**a**) and cisplatin groups (**b**) (n = 6). **c**. A proposed model illustrating the role of CAF-derived exosomal miR-522 in regulating ferroptosis in GC cells. ** indicates *p* < 0.01

The inhibited expression of USP7, hnRNPA1 and miR-522 prolonged the survival period of tumor-bearing mice (Fig. 8a), and the mice in the cisplatin groups had a better survival compared with mice treated with saline (Fig. 8b). Finally, we provide a schematic diagram to show the biological role of transcellular signaling pathway, comprising USP7, hnRNPA1, exo-miR-522 and ALOX15, in regulating ferroptosis of GC cells (Fig. 8c).

## Discussion

Ferroptosis, the novel form of non-apoptotic regulated cell death, has been characterized by the accumulation of lipid peroxidation products in a cellular-iron dependent manner [3, 39, 40]. Recent studies indicated that activation of ferroptosis related pathways effectively prevents tumor progression and enhances effects of chemo-therapy, targeted therapy and even immunotherapy [4, 7, 41]. The benign cells in microenvironment of solid tumors generally contribute to tumor development and drug resistance [42–44]; but CD8^+^ T cells are found to promote tumor ferroptosis during cancer immunotherapy in recent study [41]. However, the potential roles of the other stromal cells, especially CAFs, in regulating ferroptosis of tumor cells are still blank.

The family of arachidonate lipoxygenases is regarded as the key mediator of lipid peroxidation production and eventually lead to ferroptosis, though the other types of dioxygenases are also reported to participate in this process [28, 45, 46]. Furthermore, both the inhibition of cysteine intake by and the inactivation of the phospholipid peroxidase glutathione peroxidase 4 (GPX4) result in overwhelming lipid peroxidation that causes cell death [47, 48]. Therefore, lipid ROS maintains a dynamic balance in cancer cells to avoid ferroptosis. Previous studies have focused on the potential use of GPX4 as a therapeutic target [48, 49], but the mechanism of how ALOX15 expression is suppressed remains poorly understood. The current study suggested that CAF-derived exosomes play a key role in the regulation of ALOX15 expression and lipid-ROS production in cancer cells, proving that exosomes in tumor associated microenvironment are linked with ferroptosis for the first time.

Chemo-therapy is the main method of treatment for advanced cancers, and cisplatin and paclitaxel are the first-line chemotherapeutic drugs in GC. But resistance to cisplatin and paclitaxel has become increasingly severe in GC treatment [50–52]. Resistance to chemo-therapy is generally associated with DNA damage repair, mutations of the molecules regulating cell apoptosis and increased levels of glutathione (GSH) [53, 54]. Here, we show that changes on ferroptosis-related signaling pathway may provide a new idea to reverse chemotherapy resistance. Exosomes can promote the development of chemo-resistance in tumor cells, and the in-depth understanding of the mechanisms involved in drug resistance would contribute to improve therapeutic effects as well as prognosis. In this study, we demonstrated that the block of CAF-exosomes mediated lipid-ROS inhibition leads to increased ferroptosis levels in cancer cells, which enhanced sensitivity of chemo-therapy. Further studies are needed to learn more about the effects of ferroptosis in the other strategies of GC treatment, especially targeted therapy.

Our study suggested that USP7 promotes miR-522 secretion from CAFs through regulating deubiquitination on hnRNPA1, which is specifically involved in miR-522 packaging into exosomes. Knockdown of USP7 or hnRNPA1 sharply decreases miR-522 levels in microenvironment, causing elevated cell death and improved chemo-sensitivity. Hence, inhibiting the secretion of specific miRNA from CAFs serves as a novel method for the clinical therapy of GC.

However, none of the ferroptosis-related genes described in this study are classical tumor-promoting factors. Since miR-522 plays a key role in mediating the down-regulation of ALOX15 and lipid-ROS, resulting in suppressed cell death, we believe that miR-522 serve as the tumor-driving factor. Although USP7, hnRNPA1 and miR-522 are proved to be up-regulated in gastric cancer, they are also widely expressed in normal tissues. Given that the expression and modification of these genes are more complicated in human body, there is still a long way before these results can be used for clinical treatment.

## Conclusions

Our study illustrated the novel role of TME derived exosomes in regulating ferroptosis of cancer cells by transporting special signaling message. This study also provides new idea to trigger cellular demise in tumor by blocking specific miRNA’s packaging into exosomes.

## Supplementary information

**Additional file 1: SupplementalFigure1.** HE staining of the GC tumor tissues and para-carcinoma tissues. **Supplemental Figure2.** The expression features of USP7, hnRNPA1 and ALOX15 in GC tumor tissues. **Supplemental Figure3**. Exosomal miR-522 serves as a potential up-stream regulator of ALOX15. **Supplemental Figure4.** Purity measurement of primary tumor cells, NFs and CAFs. **Supplemental Figure5.** Screening of ALOX15 related miRNAs secreted from CAFs. **Supplemental Figure6.** miR-522 restrains erastin-induced ferroptosis in GC cells by directly targeting ALOX15. **Supplemental Figure7.** Evaluation of the proliferation of lenti-virus transduced CAFs in vivo.

## Data Availability

Not applicable.

## Abbreviations

ALOX15: Arachidonate lipoxygenase 15
CAFs: Cancer-associated fibroblasts
EVs: Extracellular vesicles
GC: Gastric cancer
GPX4: Glutathione peroxidase 4
HE: Hematoxylin-Eosin
hnRNPA1: Heterogeneous nuclear ribonucleoprotein A1
IHC: Immunohistochemistry
lipid-ROS: Lipid peroxides
MMP: Mitochondrial membrane potential
TME: Tumor microenvironment
USP7: Ubiquitin-specific protease 7
