# Supplementary material for: CAF secreted miR-522 suppresses ferroptosis and promotes acquired chemo-resistance in gastric cancer
Source: Mol Cancer. 2020 Feb 27;19:43. doi: 10.1186/s12943-020-01168-8 (PMC7045485; doi:10.1186/s12943-020-01168-8)
Supplement: Supplementary file 1 — Additional file 1: SupplementalFigure1. HE staining of the GC tumor tissues and para-carcinoma tissues. Supplemental Figure2. The expression features of USP7, hnRNPA1 and ALOX15 in GC tumor tissues. Supplemental Figure3. Exosomal miR-522 serves as a potential up-stream regulator of ALOX15. Supplemental Figure4. Purity measurement of primary tumor cells, NFs and CAFs. Supplemental Figure5. Screening of ALOX15 related miRNAs secreted from CAFs. Supplemental Figure6. miR-522 restrains erastin-induced ferroptosis in GC cells by directly targeting ALOX15. Supplemental Figure7. Evaluation of the proliferation of lenti-virus transduced CAFs in vivo. [file 12943_2020_1168_MOESM1_ESM.docx]

**
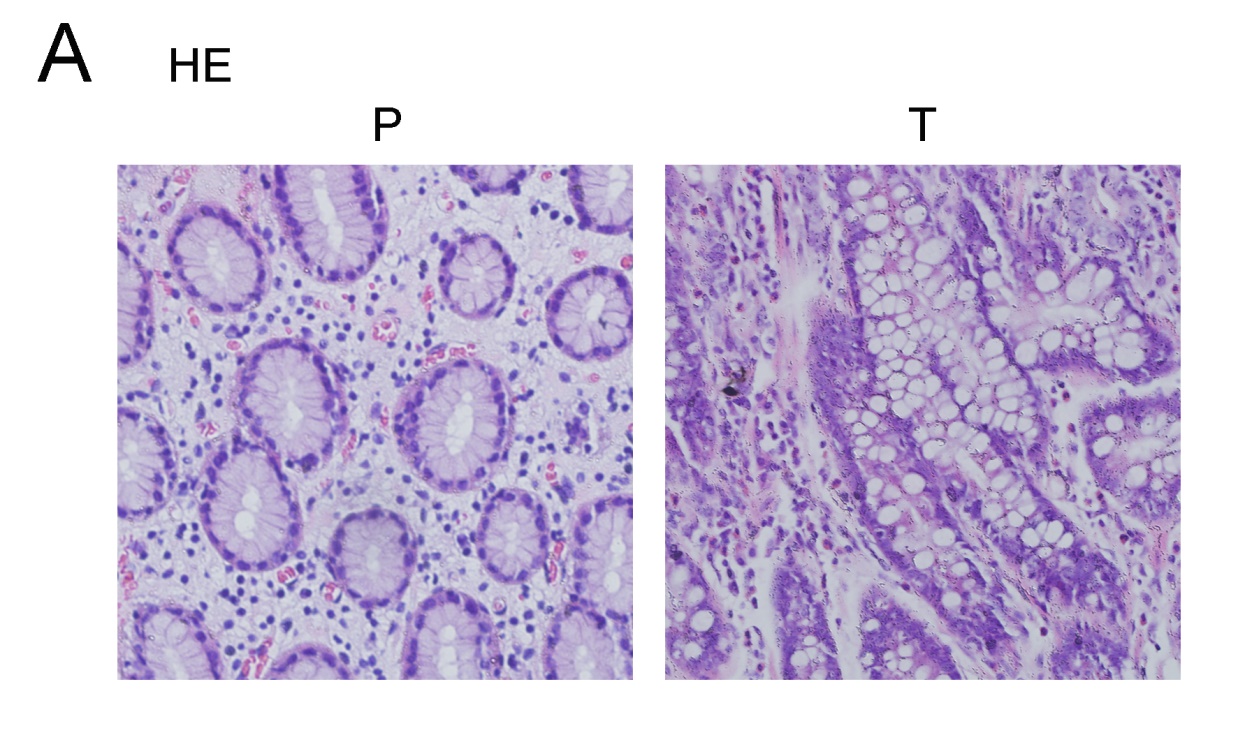
**

**Supplemental Figure 1. HE staining of the GC tumor tissues and para-carcinoma tissues.** A. The paired tumor tissues (T) and para-carcinoma (P) described in Figure 1 B were checked by hematoxylin-eosin (HE) staining (n=12).


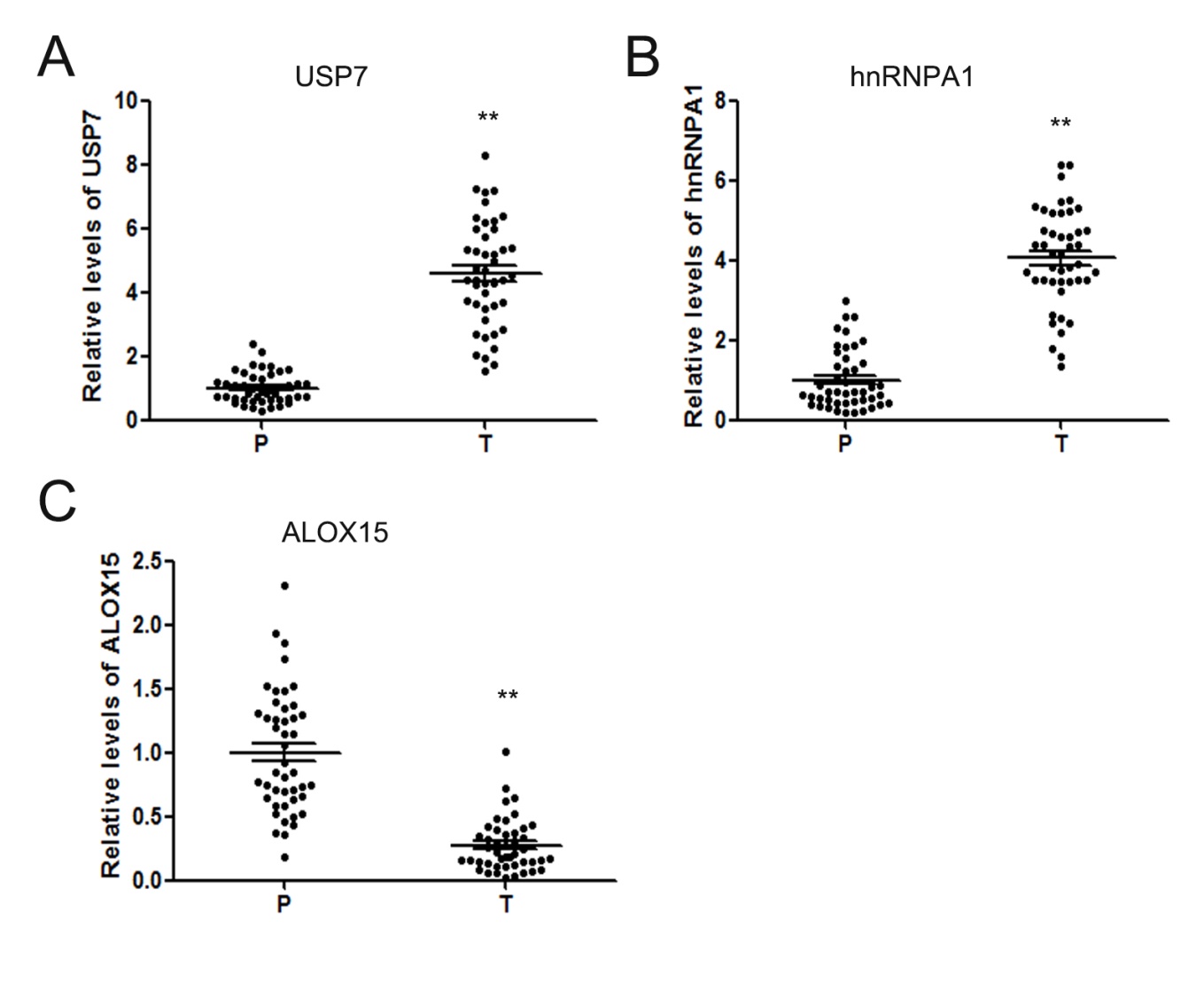


**Supplemental Figure 2. The expression features of USP7, hnRNPA1 and ALOX15 in GC tumor tissues.** The relative levels of USP7 (A), hnRNPA1 (B) and ALOX15 (C) in tumor tissues (T) and paired para-carcinoma tissues (P) (n=45). ** indicates p < 0.01.

**
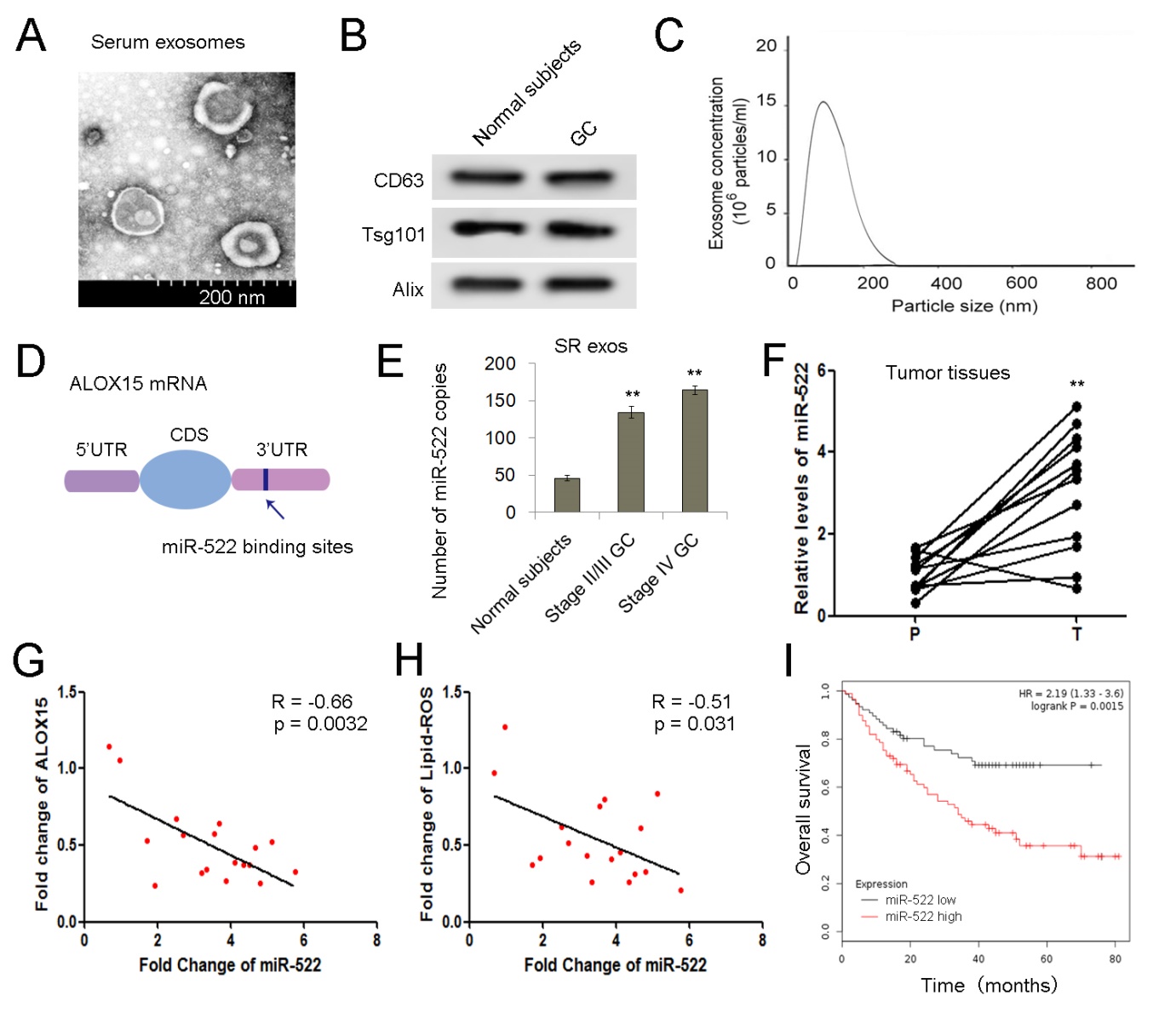
**

**Supplemental Figure 3. Exosomal miR-522 serves as a potential up-stream regulator of ALOX15.** A. TEM image of exosomes isolated from human plasma (scale bar, 200 nm). B. Exosomes were isolated from the serum of both normal subjects and GC patients. Western blotting of three representative exosome-specific markers: CD63, Alix, and Tsg101. C. NTA analysis showing the size range of plasma exosomes. D. The predicted miR-522 binding region in the 3’UTR of ALOX15 mRNA. E. Number of miR-522 copies in serum exosomes of normal subjects, stage II/III and stage IV GC patients (n=150). F. Relative levels of miR-522 in gastric tumor tissues (n=12). (G-H) Analysis of the interaction between miR-522 and ferroptosis, and miR-522 is negatively related with both ALOX15 expression (G) and lipid-ROS levels (H) (n=20). I. Analysis of overall survival in miR-522 high (n=184) and miR-522 low groups (n=162). ** indicates p < 0.01.

**
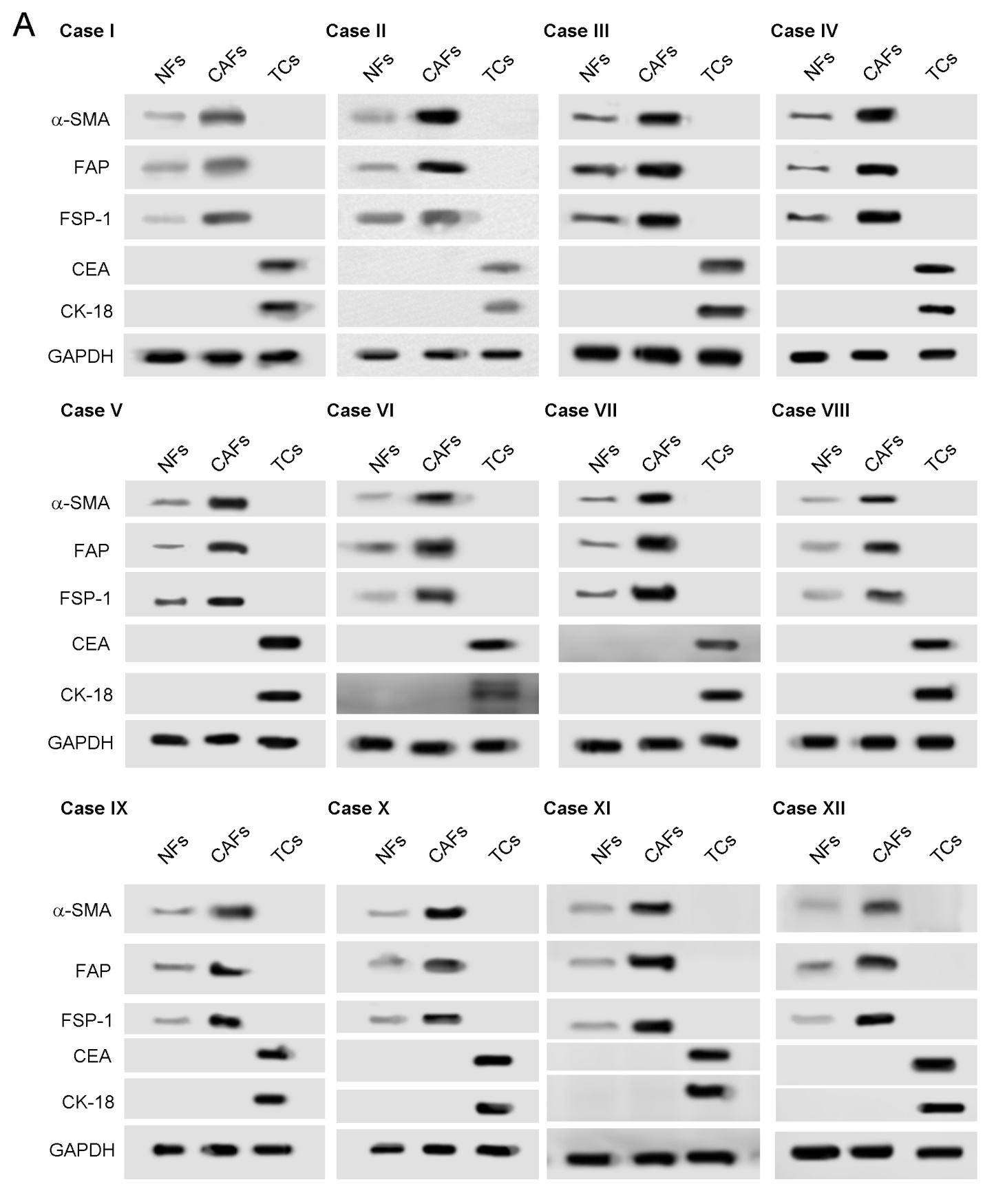
Supplemental Figure 4. Purity measurement of primary tumor cells, NFs and CAFs.** A. WB analysis of α-SMA, FAP, FSP-1, CEA and CK-18 in NFs, CAFs and TCs (n=12).


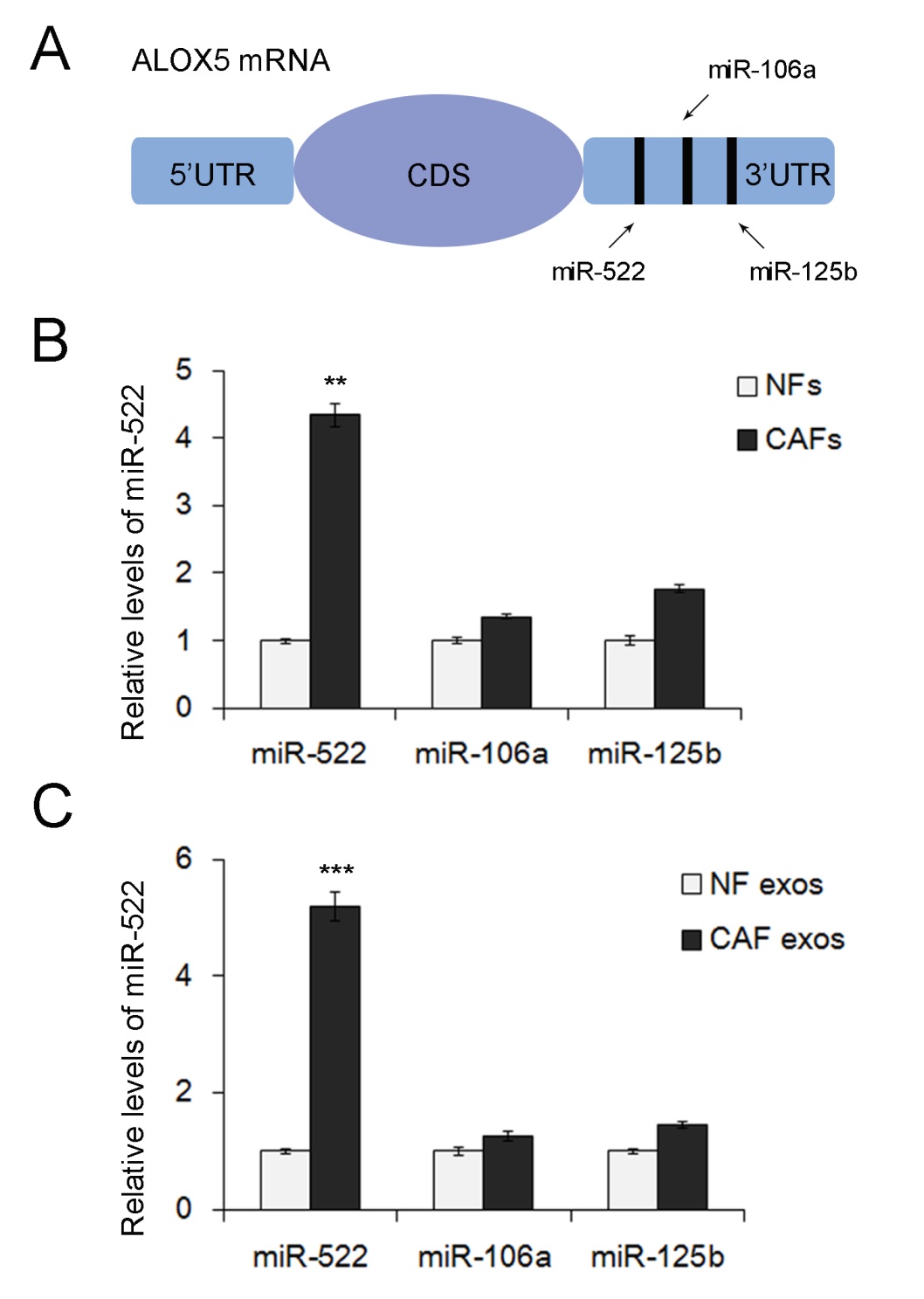


**Supplemental Figure 5. Screening of ALOX15 related miRNAs secreted from CAFs.** A. The three predicted up-stream miRNAs of ALOX15. B. Quantification of miR-522, miR-106a and miR-125b in paired NFs and CAFs (n=12). C. RT-qPCR analysis of miR-522, miR-106a and miR-125b in the exosomes of NFs and CAFs (n=12). ** indicates p < 0.01; *** indicates p < 0.001.


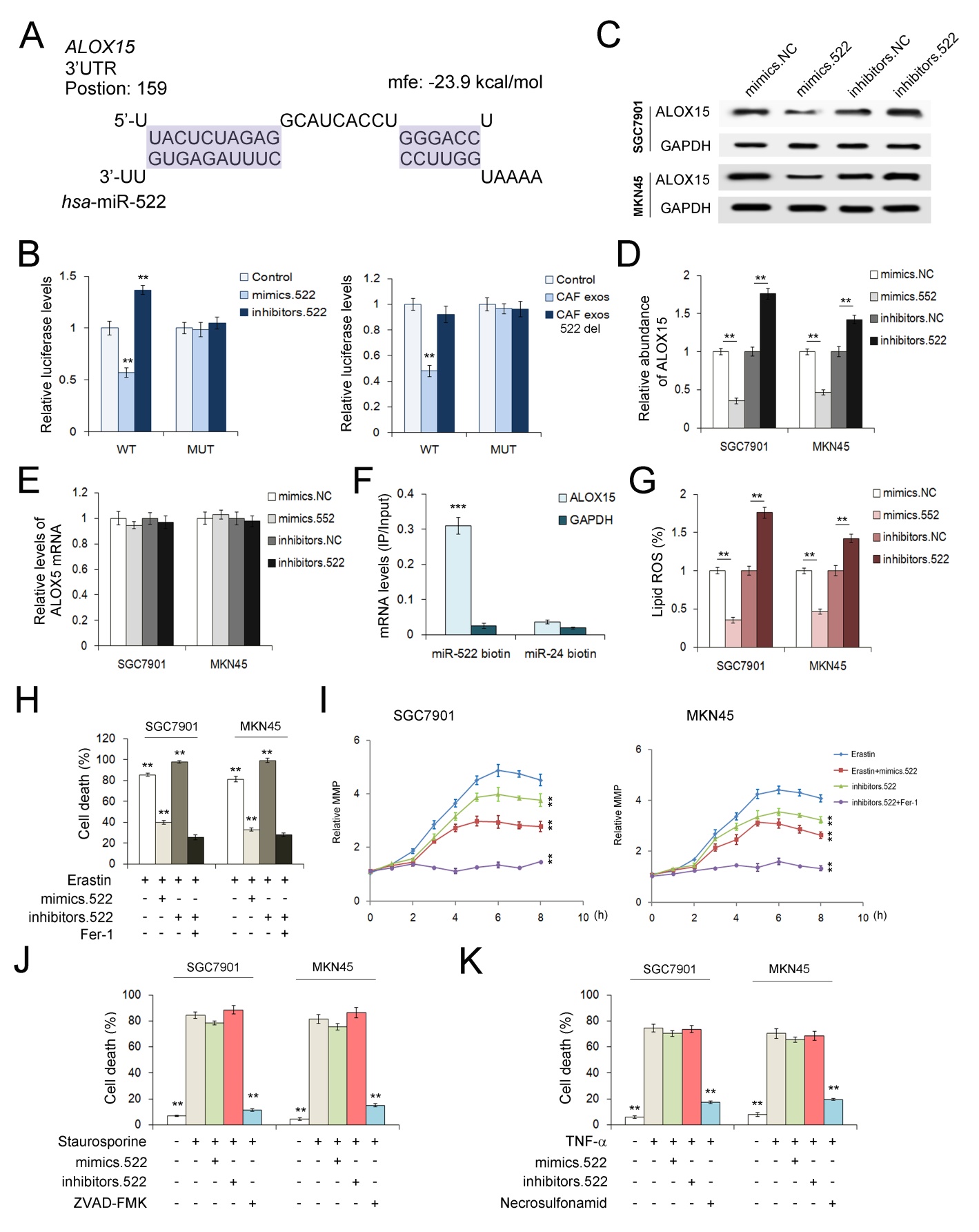


**Supplemental Figure 6. miR-522 restrains erastin-induced ferroptosis in GC cells by directly targeting ALOX15.** A. Predicted binding sites of miR-522 within the 3’-UTR of ALOX15 mRNA. B. Direct recognition of ALOX15 3’-UTR by miR-522 (n=3). SGC7901 cells were co-transfected with firefly luciferase reporters containing either wild-type or mutant (mut) ALOX15 3’-UTR with miR-522 mimics, inhibitors and the corresponding normal control. The relative luciferase levels were detected using a luciferase kit. C. WB analysis of ALOX15 in SGC7901 cells and MKN45 cells treated with miR-522 mimics or inhibitors (n=3). D. Quantitative analysis of (C) (n=3). E. Relative levels of ALOX15 mRNA in GC cells treated as described above (n=3). F. RT-qPCR analysis of ALOX15 mRNA captured by biotin-miR-522 (n=3). (G-I) miR-522 suppresses erastin-induced ferroptosis in GC cells. Effects of miR-522 mimics/inhibitors, erastin and Fer-1 on lipid-ROS production (G), cell death (H) and MMP (I) (n=3). J. Indicated GC cells were treated with apoptosis inducer staurosporine (0.5 μM) and miR-522 with or without ZVAD-FMK (10 μM) and cell death ratio was assayed (n=3). K. The cell death ratio in GC cells treated with necroptosis inducer (TNF-α) (10 nmol/ml) and miR-522 with or without necrosulfonamide (0.5 μM). ** indicates p < 0.01.


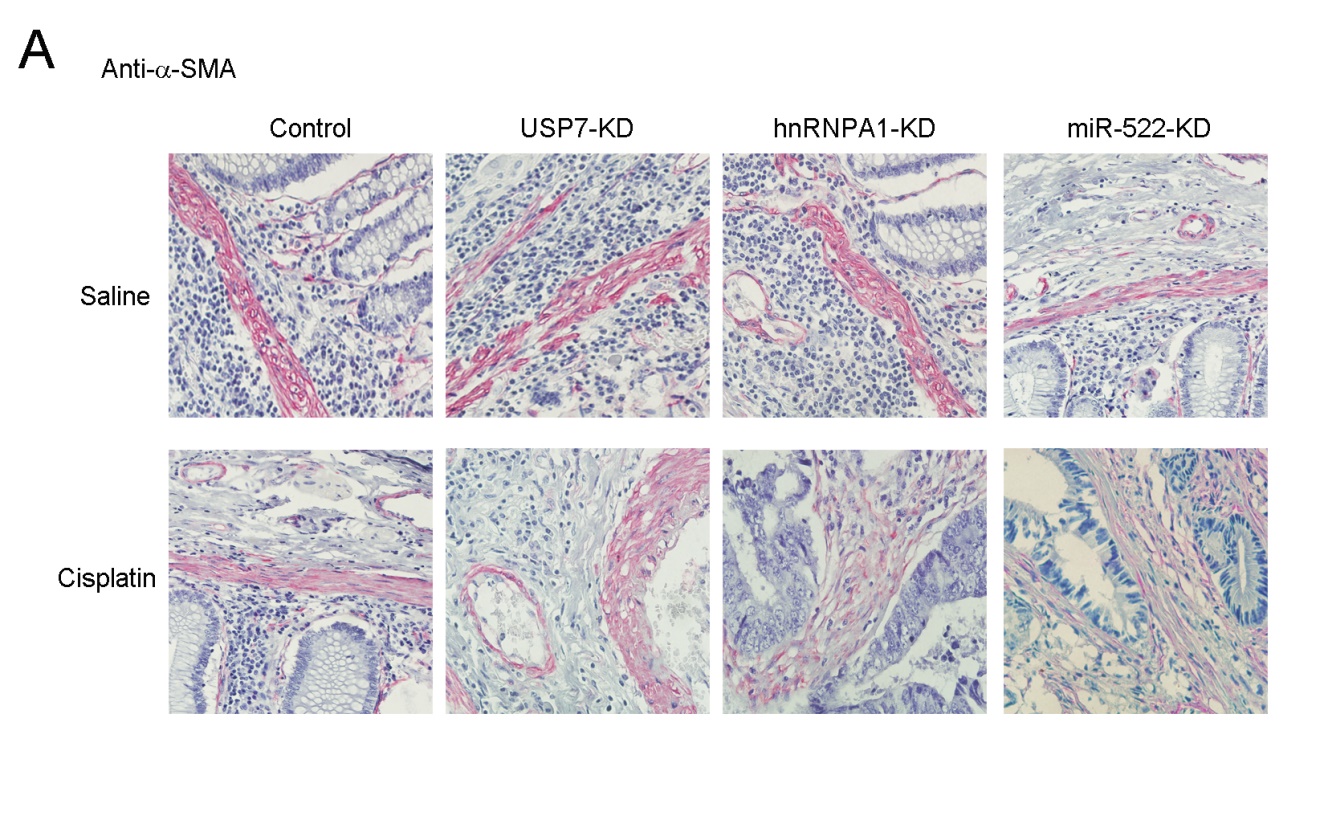


**Supplemental Figure 7. Evaluation of the proliferation of lenti-virus transduced CAFs in vivo.** A. IHC analysis of α-SMA in the tumor tissues described in Figure 9A (n=6).
